# Prevalence of spinal pain in a population of Bosnia and Herzegovina

**DOI:** 10.3934/publichealth.2022053

**Published:** 2022-12-05

**Authors:** Antonija Hrkać, Roberta Perković, Branko Krišto, Livia Puljak

**Affiliations:** 1 Faculty of Health Studies, University of Mostar, Mostar, Bosnia and Herzegovina; 2 Cantonal Hospital, Dr. Fra Mihovil Sučić, Livno, Bosnia and Herzegovina; 3 School of Medicine, University of Mostar, Mostar, Bosnia and Herzegovina; 4 Center for Evidence-Based Medicine and Health Care, Catholic University of Croatia, Zagreb, Croatia

**Keywords:** spinal pain, back pain, neck pain, thoracic pain, low back pain, prevalence

## Abstract

**Objectives:**

To determine the prevalence and risk factors of spinal pain in the population of Bosnia and Herzegovina (BiH).

**Methods:**

This was a cross-sectional survey conducted online in November/December 2018. Participants were inhabitants of BiH of both sexes, aged ≥ 16 years. The sample was stratified based on region and demographic characteristics. Current pain was analyzed; point prevalence was measured.

**Results:**

We received 1048 responses, of which data from 1017 could be used. The prevalence of spinal pain in BiH was 70.9%: 75.5% in women (n = 440) and 64.7% in men (n = 281). Low back pain (LBP) was more common compared to neck pain (NP) and thoracic pain (TP) in both sexes and all age groups. Significant associations with spinal pain in the bivariate analysis were found for the following groups: women, aged from 30 to 50 years, with high school education, employed persons and retirees, spinal pain in parents, smoking, irregular physical activity, longer use of TV or computer/mobile phone per day.

**Conclusion:**

To our knowledge, this is the first study of the prevalence of spinal pain in BiH. Some factors associated with spinal pain are modifiable. Therefore, public health interventions should target those factors to reduce the burden of spinal pain in BiH.

## 1. Introduction

Spinal pain has been a global public health problem for decades [1]. Depending on the segment of the spine in which the pain appears, spinal pain is divided into neck pain (NP), thoracic pain (TP) and low back pain (LBP). NP is defined as the pain localized in the anatomic region of the neck (from the superior nuchal line to the spine of the scapula, from the external occipital protuberance to the superior border clavicle and the suprasternal notch), with or without pain radiating to the head, trunk and upper limb (s). TP is defined as spinal pain with localization from the cervical–thoracic hinge (C7–T1) to the thoracic–lumbar junction (T12–L1). LBP is defined as pain, muscle tension and restricted mobility, typically between lower rib margins and inferior gluteal folds, with or without radiating pain to one or both legs [2]–[4]. LBP and NP are the more common spinal pains and the leading causes of population disability, regardless of sex and age [1],[5]. TP has been less studied, although this type of spinal pain is equally disabling and burdensome for the individual, community and work environment [6],[7]. Regardless of spinal pain type, the symptoms, etiology and consequences are generally the same. Potential risk factors that are associated with spinal pain are demographic, hereditary, lifestyle and occupational factors [8]–[10].

Global epidemiological studies have reported that point-prevalence for LBP ranged from 1.0% to 58.1%, for NP ranged from 0 to 41.5% and for TP ranged from 4.0% to 72.0% [6],[11],[12]. In 19 European countries, the estimated one-year prevalence of LBP/NP ranged from 16.08 to 54.05% [13]. However, it was pointed out that the exact prevalence of spinal pain is difficult to determine due to the heterogeneity of the study methods, various definitions of spinal pain and data collection methods used by researchers [11],[12]. Moreover, several studies reported that many countries and regions lack data on population-based prevalence, risk factors and burden estimates for all three types of spinal pain [1],[5],[7]. The lack of prevalence data is particularly pronounced for the countries of the former Balkans, the geographical location of Bosnia and Herzegovina (BiH) [14]. Moreover, BiH was not included in the spinal pain prevalence European study, and we were unable to find such data in the published literature [13].

Determining the exact prevalence of LBP and other types of spinal pain is important for many reasons for experts in this field. Pain prevalence studies enable the assessment of burden and are important for developing effective public health policies, as well as changing existing public health policies. Prevalence data may foster health experts in this field and research aimed at improving diagnosis and treatments. Additionally, knowledge of the exact disease prevalence can lead to changes in curricula for educating medical experts such as physiotherapists and nurses in terms of providing more study hours for the most prevalent conditions in this profession [15]. This study aimed to determine the prevalence and risk factors for spinal pain in BiH among the general population via an online questionnaire.

## 2. Materials and methods

### 2.1. Study design

This was a cross-sectional observational web-based survey. The design of the study was based on previous studies [16],[17]. An online survey using Google Forms (https://docs.google.com/forms/u/0/) was created and sent by multiple channels to contacts and groups of various organizations located across BiH (both health and non-health organizations). The invitation to participate in the study was published two weeks before the start of the study via the social networks Facebook, email, WhatsApp and Viber; the same call with a link to the survey questionnaire was repeated at the beginning of the study.

We used a non-probabilistic sample stratified based on the geopolitical regions of BIH and demographic characteristics. The data were collected in November and December 2018, and the study was completed in January 2019.

The study was approved by the Ethics Committee of the Faculty of Health Studies, University of Mostar, Mostar, BIH (approval number: 01-994/18 from October 19, 2018). In the introductory part of the questionnaire and the cover letter, the participants were informed about the study, their rights and that the completion of the survey will be considered their informed consent.

### 2.2. Participants

Inclusion criteria were as follows: residents of BIH, aged ≥16 years of both sexes, having an email, Facebook, WhatsApp or Viber account for survey receipt.

Exclusion criteria were as follows: age ≤15 years, persons not residing in BIH (if participants did not provide information about BIH canton of residence, they were excluded), missing data (we excluded participants with more than half missing answers in the questionnaire), discrepant data on the presence of spinal pain (in one part of the questionnaire the participants indicated that they had spinal pain, and in the other part of the questionnaire that they did not have it) and duplicates (multiple submissions with identical time zone data and personal data of participants).

### 2.3. Questionnaire

For the study, we designed a questionnaire based on a literature review [16],[18]. Pilot testing of the questionnaire for understanding, clarity and relevance was conducted before the start of the survey. It consisted of 29 questions about demographic characteristics and spinal pain. A detailed description and the full version of the questionnaire are in Appendix 1. To avoid recall bias, we asked participants about their present pain and its duration. The point prevalence was measured.

### 2.4. Data collection

Potential participants received a personal message (cover letter) with a written invitation about the study and a link to the study. Details are described in Appendix 1. Only the principal investigator (AH) had access to the stored data (Appendix 1).

### 2.5. Sample size

The sample size was determined by using computer software. According to the latest census data about the population in BIH from 2013, BIH had 2,987,440 inhabitants over 14 years of age in BIH. Based on the 95% confidence level, the population proportion of 50% and absolute precision of 5%, it was determined that 385 participants were needed. Assuming that the difference in the established prevalence between persons with spinal pain and those who do not have spinal pain is 10%, with an alpha value of 0.05%, a beta value of 0.2% and the power of study of 0.8, we calculated that a total of 782 participants were needed. However, to satisfy the study criteria, the representation of participants by geographical and demographic strata, and to improve the quality of the study, we decided to increase the sample size to meet as closely as possible the percentage of the BIH population according to the data of the 2013 Census.

### 2.6. Statistical methods

Sample weighting was performed before the start of data analysis by the variables of region, sex and age group. Study weight was ensured by stratification of participants based on geopolitical regions of BiH and demographic characteristics (sex, age). The required percentages for the specified variables were calculated by data from the 2013 BIH Census. The determined frequencies were converted into percentages; based on them, sample balancing was performed. Analyses of the results were performed through weighted and unweighted estimates, and due to the absence of significant differences, they were presented without weighted variables. The normality of the data distribution was tested by visual inspection of the histogram. Descriptive categorical variables were presented as frequencies and percentages, and quantitative variables were presented as mean and standard deviation. The significance of the difference between the categorical variables was analyzed by the Chi-square test. Quantitative variables' differences between the two groups were tested by the Independent Student t-test, and differences between the three groups were tested by one-way ANOVA. The association of factors with the presence of spinal pain was conducted by logistic regression through bivariate and multivariate analysis. Multivariate analysis was conducted through three models: Model I consisted of demographic and occupation factors; Model II consisted of demographic, occupational and hereditary factors; and Model III consisted of demographic, occupational, hereditary and behavioral factors. The significance of the differences in the regression analyses was tested by Wald's Chi-square test, and the results were presented as the prevalence ratio and 95% confidence interval of the prevalence ratio. The statistical significance was p < 0.05 in all analyses. Statistical software IBM SPSS Statistics 23 (Armonk, NY: IBM Corp.) was used for data analysis.

## 3. Results

### 3.1. The sample

The total number of sent invitations was 1682 (email n = 705, text messages n = 774, Facebook n = 203), and the total number of collected responses was 1048. From 1048 study responses, we excluded 3% of surveys (n = 31) due to inadequate age (n = 10), missing data (n = 7), false responses (n = 3), discrepant data (n = 5) and duplicates (n = 6).

The final analysis included 1017 participants, of both sexes, aged from 16–78 years. The majority of participants were women (57%), aged 30–50 years, with a high-school education. Detailed socio-demographic characteristics of the participants are shown in Table 1.

**Table 1.** Socio-demographic characteristics of the surveyed population of Bosnia and Herzegovina.

Variables		Men (N = 434)	Women (N = 583)	Total (N = 1017)
Age^†^		45.6 (15.0)	38.1 (11.4)	41.3 (13.5)
Age group 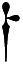				
	16–29 years	62 (14.3)	142 (24.4)	204 (20.1)
	30–50 years	196 (45.2)	358 (61.4)	554 (54.5)
	≥51years	176 (40.6)	83 (14.2)	259 (25.5)
Height^†^		182.6 (6.1)	170.4 (5.8)	175.6 (8.5)
Weight^†^		90.1 (12.7)	70.0 (11.2)	78.6 (15.5)
BMI^†^		27.0 (3.4)	24.1 (3.7)	25.3 (3.9)
BMI classification 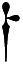				
	Underweight	- (-)	12 (2.1)	12 (1.2)
	Normal range	134 (30.9)	378 (64.8)	512 (50.3)
	Overweight	216 (49.8)	149 (25.6)	365 (35.9)
	Obese class I	84 (19.3)	44 (7.5)	128 (12.6)
	Obese class II	- (-)	- (-)	- (-)
	Obese class III	- (-)	- (-)	- (-)
Educational level 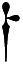				
	Students	10 (2.3)	29 (5)	39 (3.8)
	Elementary school	1 (0.2)	3 (0.5)	4 (0.4)
	High school	218 (50.2)	310 (53.2)	528 (51.)
	College	205 (47.2)	241 (41.3)	446 (43.9)
Employment status 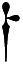 (N = 978)^‡^				
	Employed	356 (36.3)	486 (49.6)	842 (85.9)
	Unemployed	19 (2)	54 (5.5)	71 (7.5)
	Retired	51 (5.1)	14 (1.5)	65 (6.6)

*Note: BMI: Body mass index; †mean (standard deviation); 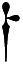 n (%); ‡ Without students.

A comparison of the sample with the BiH population based on the 2013 BiH Census demonstrated that they were similar (data not shown) for sex and regional residence. Age was also similar, although the 30 to 50 age group was slightly overrepresented, and the ≥51 age group was slightly underrepresented in the sample.

### 3.2. Prevalence of spinal pain syndrome

**Figure 1.**
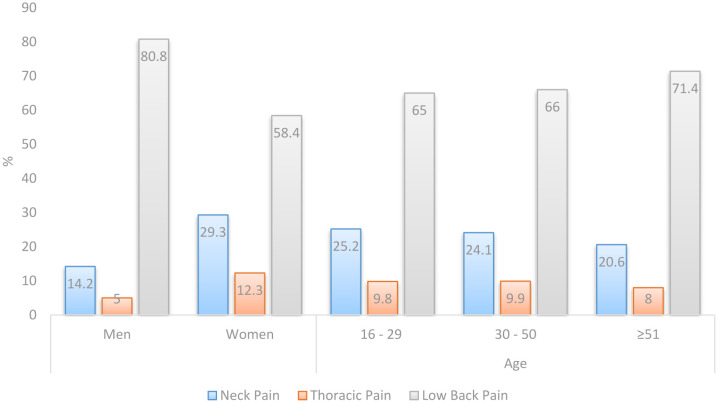
Prevalence of spinal pain with regard to anatomic locations by sex and age group in the surveyed population of Bosnia and Herzegovina.

The presence of global spinal pain was reported by 721 participants (70.9%): 440 (75.5%) women and 281 (64.7%) men (Table 2). Of the total number of reported spinal pain, 484 (67.1%) participants had LBP, 169 (23.4%) participants had NP, and 68 (9.4%) participants had TP. Women reported more NP and TP, and men reported more LBP. The prevalence of spinal pain, depending on the anatomical location, is shown in Figure 1.

**Table 2.** Point prevalence of spinal pain in surveyed population in Bosnia and Herzegovina.

Variables		Total N = 1017	Spinal pain N = 721 N (%)	PR (95% CI of PR)
Sex	Men	434	281 (64.7)	1
	Women	583	440 (75.5)	1.67 (1.27–2.20)*
Age	16–29	204	123 (60.3)	1
	30–50	554	423 (76.4)	2.13 (1.51–3.0)*
	≥51	259	175 (67.6)	1.37 (0.94–2.01)
BMI	Underweight	12	4 (33.3)	1
	Normal range	512	355 (69.3)	4.52 (1.34–15.24)***
	Overweight	365	271 (74.2)	5.77 (1.70–19.6)**
	Obese class I	128	91 (71.1)	4.92 (1.40–17.3)***
Education	Student	39	22 (56.4)	1
	Elementary school 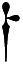	4	4 (100)	- (-)
	High school	532	396 (74.4)	2.23 (1.15–4.32)***
	College	446	303 (67.9)	1.64 (0.84–3.18)
Work	Student 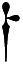	39	38 (97.4)	- (-)
	Unemployed	71	34 (47.9)	1
	Employed	842	605 (71.9)	2.78 (1.70–4.53)*
	Retired	65	44 (67.7)	2.28 (1.14–4.58)***
Type of work (N=844)	Sedentary	553	395 (71.4)	1
	Physical	291	228 (78.4)	1.45 (1.04–2.02)***
Hereditary (SP in parents)	No	444	287 (64.6)	1
	Yes	573	434 (75.7)	1.71 (1.30–2.24)*
Smoking	No	712	491 (69)	1
	Yes	305	230 (75.4)	1.38 (1.02–1.87)***
Physical activity (PA)	No	299	210 (70.2)	1
	Yes	718	511 (71.2)	1.05 (0.78–1.41)
Frequency of PA (day/week) (N = 718)	1–2	217	146 (67.3)	1
	3–4	191	123 (64.4)	0.88 (0.58–1.51)
	5–6	117	77 (65.8)	0.94 (0.52–1.51)
	Irregular	193	165 (85.5)	2.87 (1.75–4.68)*
TV (Hours/day)	1–2	632	429 (67.9)	1
	2–3	297	226 (76.1)	1.51 (1.10–2.06)***
	4–5	88	292 (75.8)	1.42 (0.85–2.37)
Computer/Mobile phone (Hours/day)	1–2	333	252 (75.7)	1
	3–4	273	189 (69.2)	0.72 (0.50–1.03)
	5–6	411	280 (68.1)	0.69 (0.50–0.95)***

*Note: PR (95% CI): Prevalence ratio (95% confidence interval of prevalence ratio); BMI: Body mass index; *Statistical significance p < 0.001; **Statistical significance p < 0.01; ***Statistical significance p < 0.05; 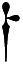
Excluded from analysis.

In bivariate regression, significant associations with the presence of spinal pain were found for the following groups: women, aged 30 to 50 years, with high school education, employed and retired persons, physical work, smoking, irregular physical activity, watching TV 2 to 3 hours per day and use of a computer or cell phone 5 to 6 hours on a day. Likewise, the bivariate analysis found a statistically significant difference between normal and elevated body mass index, but due to the excessive range of the confidence interval, this result is questionable. No statistically significant difference was found in the repeated analysis without underweight body mass index (Table 2). Multivariate analysis confirmed statistically significant associations with the presence of spinal pain for the following groups: women, aged 30 to 50 years, employed and retired persons, spinal pain in parents and irregular physical activity (Table 3).

### 3.3. Characteristics of pain

**Figure 2.**
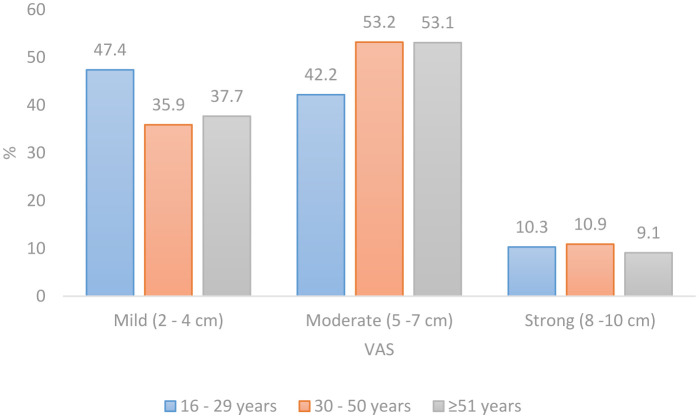
Distribution of pain intensity expressed with visual analog scale (VAS) by age group in the surveyed population of Bosnia and Herzegovina.

The average intensity of current spinal pain on a scale from 0 to 10 was 5, without difference by sex, but a significant difference was found in the pain intensity score in different age groups (F (2) = 4.562; p = 0.011). Characteristics of spinal pain by sex are shown in Table 4. The mean pain intensity in the age group from 16 to 29 years was 4.6 (SD 2.0), in the age group from 30 to 50 years was 5.2 (SD 1.8) and in the age group ≥51 years was 5.2 (SD 1.2). More than half of the participants in the age group 30 to 50 years, as well as 51 years, reported moderate pain intensity (53.2% vs. 53.1%), while participants in the age group 16 to 29 years reported a higher percentage of mild pain (47.4%) (Figure 2).

**Table 3.** Multivariate analysis for the independent demographics, occupational, hereditary and behavioral factors associated in bivariate analysis (p < 0.02) with spinal pain in a surveyed population of Bosnia and Herzegovina.

Variables		Model I PR (95% CI)	Model II PR (95% CI)	Model III PR (95% CI)
Sex				
	Men	1	1	1
	Women	1.64 (1.21–2.21)**	1.60 (1.18–2.16)**	1.32 (0.89–1.96)
Age			
	16–29	1	1	1
	30–50	2.01 (1.36–2.99)*	2.17 (1.45–3.25)*	2.39 (1.45–3.92)**
	≥51	1.45 (0.90–2.33)	1.70 (1.04–2.78)^c^	1.18 (0.98–1.44)
Education				
	Students	1	1	1
	High school	1.54 (0.74–3.20)	1.62 (0.78–3.39)	1.48 (0.62–3.52)
	College	1.08 (0.51–2.83)	1.09 (0.51–2.33)	0.99 (0.41–2.43)
	Employment status		
	Unemployed	1	1	1
	Employed	2.85 (1.71–4.75)*	3.15 (1.87–5.31)*	3.53 (1.84–6.76)*
	Retired	2.91 (1.32–6.42)**	2.98 (1.34–6.65)**	1.60 (1.09–2.35)***
Spinal pain in parents		
	No		1	1
	Yes		1.86 (1.39–2.49)*	1.71 (1.19–2.45)**
Frequency of physical activity			
	1–2 days/week			1
	3–4 day/week			1.04 (0.67–1.61)
	5–6 days/week			1.19 (0.70–2.02)
	Irregular			2.88 (1.70–4.87)*
	Time watching TV			
	1–2 hours/day			1
	3–4 hours/day			1.48 (0.99–2.23)
	4–5 hours/day			2.01 (0.92–4.38)

*Note: PR: Prevalence ratio; 95% CI: 95% confidence interval; *Statistical significance p < 0.001; **Statistical significance p < 0.01; ***Statistical significance p < 0.05.

The mean pain intensity in participants who reported LBP and NP was 5.1 (SD 1.8 vs. 1.9), and in TP it was 4.8 (SD 1.5). The presence of pain up to 6 weeks was reported by 78 (46.2%) participants with NP, 36 (52.9%) with TP and 194 (40.1%) participants with LBP. Pain present for more than 12 weeks was reported by 74 (43.8%) participants with NP, 30 (44.1%) participants with TP and 257 (53.1%) participants with LBP.

### 3.4. Consequences of global spinal pain syndrome

Diagnosed changes in the spine were confirmed by 40% of the participants; women had such a diagnosis more commonly than men (44% vs. 34%; p < 0.05). About 54% of participants sought the help of health professionals to relieve spinal pain; most commonly, they visited physiotherapists (52%). Medication use for the alleviation of spinal pain was confirmed by 34% of participants (Table 4).

Knowledge of good posture during various daily activities and its importance in the prevention and treatment of spinal pain was reported by 71% of participants; men's affirmative answers were significantly higher than women's (77% vs. 66%). However, the use of good posture in everyday life was reported by only 25% of the participants (Table 4).

Men were significantly longer on sick leave because of spinal pain compared to women (p < 0.001); absence from work or school for 3 ≥ days was confirmed by 72% of men, and 54% of women confirmed absence from work or school lasting from 1 to 2 days (Table 4).

**Table 4.** Characteristics and consequences of the presence of spinal pain by sex among a surveyed population of Bosnia and Herzegovina.

Variables		Total (N = 721) N (%)	Men (N = 281) N (%)	Women (N = 440) N (%)	p^ 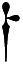 ^
Pain intensity (0–10 cm)^†^		5.1 (1.8)	4.9 (1.7)	5.2 (1.8)	0.066^‡^
	Mild (2–4)	272 (38.2)	118 (42.6)	154 (35.4)	
	Moderate (5–7)	366 (51.4)	136 (49.1)	230 (52.9)	0.097
	Strong (8–10)	74 (10.4)	23 (8.3)	51 (11.7)	
Duration of pain					
	Up to 6 weeks	308 (42.5)	127 (45.2)	181 (41.1)	
	6–12 weeks	52 (7.2)	23 (8.2)	29 (6.6)	0.307
	≥12 weeks	361 (49.8)	131 (46.6)	230 (52.3)	
Localization of pain					
	Stays in the region back	521 (72.3)	203(72.2)	318 (72.3)	0.993
	Spreads down the limbs	200 (27.7)	78(27.8)	122 (27.7)	
Frequency of pain					
	Daily	268 (37.1)	82 (29.2)	186 (42.3)	
	Once a week	104 (14.4)	42 (14.9)	62 (14.1)	0.001
	Once a month	73 (10.1)	27 (9.6)	46 (10.5)	
	After severe activity	276 (38.3)	130 (46.3)	146 (33.2)	
Diagnostic (Herniated IVD)		289 (40.1)	96 (34.2)	193 (43.9)	0.010
Medical help		387 (53.7)	144 (51.2)	243 (55.2)	0.296
Type of medical help					
	Family medicine	47 (12.1)	12 (8.3)	35 (14.3)	0.126
	Neurologist/Neurosurgeon	122 (31.4)	54 (37.5)	68 (27.9)	
	Physiotherapy	203 (52.3)	73 (50.7)	130 (53.3)	
	Someone else	16 (4.1)	5 (3.5)	11 (4.5)	
Medication use		247 (34.3)	86 (30.6)	161 (36.6)	0.099
Medication frequency					
	Daily	50 (20.2)	13 (15.1)	37 (23)	
	Once a week	53 (21.5)	14 (16.3)	39 (24.2)	0.084
	Once a month	40 (16.2)	14 (16.3)	26 (16.1)	
	Rarely	104 (42.1)	45 (52.3)	59 (36.6)	
Knowledge of proper posture* (YES)		508 (70.5)	216 (76.9)	292 (66.4)	0.003
Use of proper posture (YES)		183 (25.4)	97 (34.5)	86 (19.5)	<0.001
Absence from work/school		246 (34.1)	95 (33.5)	151 (34.6)	0.760
Frequency of absence					
	1–2 day	110 (44)	26 (28)	84 (53.5)	
	3–4 day	47 (18.8)	18 (19.4)	29 (18.5)	<0.001
	5–7 day	26 (10.4)	14 (15.1)	12 (7.6)	
	More than a week	67 (26.8)	35 (37.6)	32 (20.4)	

*Note: IVD: Intervertebral disk; ^†^Mean (standard deviation); ^
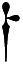
^Chi-Square test; ^‡^Independent student t-test; *Proper posture in daily activities (standing, walking, sitting, lifting and walking).

## 4. Discussion

The overall prevalence of spinal pain in the surveyed population of Bosnia and Herzegovina at the end of the year 2018, in a sample of the population aged 16–78 years, was 70.9%. LBP was the most common, with a prevalence of 67.2%, followed by NP (23.4%) and TP (9.4%). Women had a larger prevalence of NP and TP compared to men, while men had a larger prevalence of LBP (80.8% versus 58.4%). The largest prevalence of NP was found in participants aged 30 and 50 years, and LBP in those aged ≥51 years, while the prevalence of TP was found to be the same at age 50 and slightly lower at age ≥51 but with no statistical significance.

A systematic review from 2019 (Fatoye and colleagues; n = 13 studies) reported that real-world prevalence for LBP ranged from 1.4% to 20.0%. However, these data were determined based on studies from different regions/countries, which are geographically sufficiently distant from our region [19]. In our study, the determined point-prevalence for NP and TP is in line with the estimated ranges of global point prevalence, while the determined point-prevalence for LBP is higher than the global point-prevalence range [6],[11],[12]. The high prevalence of LBP and NP among our population is congruent with the results of a study published in 2019, which processed data on pain syndromes from several European countries, geographically closer to our country [13].

A lower incidence of TP compared to other spinal pain was also found in a study by Fouquet et al., which was published in 2015 and conducted in France between 2002 and 2005 among 3710 workers aged 20 to 59 years, as well as a higher susceptibility of women to TP [3]. In a systematic review by Briggs et al., which was published in 2009 and included 33 studies, the authors reported that the established prevalence point for TP ranges from 4–72% and that the higher prevalence of TP was found in childhood and adolescence, and especially in women [6]. These results are consistent with the findings of our study regarding the established prevalence and higher prevalence in women, except for the difference in prevalence based on age group, because we did not find differences in the prevalence of TP across age groups. However, in our study, we did not include participants younger than 16 years.

Sex at the risk factor associated with spinal pain in our study is in line with the evidence of previously published studies [8],[10]–[12]. In our study, women were more susceptible to NP and TP, and men to LBP. A systematic review published in 2012 reported that the average prevalence of LBP in one year was 37%, the highest in middle age and more common in women. The median overall prevalence of LBP was higher among women in all age groups; women had a significantly higher point and a one-month prevalence, while in the one-year and lifetime prevalence, there was no significant difference between the sexes [20]. On the other hand, the systematic review by Fatoye et al., which was published in 2019, reported that the male sex had a significant association with LBP compared with women [19]. The 2010 Global Report also reports greater susceptibility of men to LBP compared to women [21]. Therefore, it appears that the research literature is still ambivalent regarding the susceptibility of LBP between sexes.

In a cross-sectional study conducted in Spain between June 2006 to June 2007, which included 29,478 individuals aged ≥16, a higher prevalence of NP and LBP was found in women compared to men; the prevalence of NP was higher in individuals aged 51–70 years, and LBP in those aged >70 years [22]. The findings of that Spanish study were partially consistent with those of our study. Our findings on the association of women with the onset of NP, as well as the peak of NP prevalence in middle age, were also reported by other studies [23],[24].

In addition to sex, significant associations with spinal pain in our study were identified for occupational factors, hereditary factors, irregular physical activity, watching TV ≥3 hours per day and using a computer or mobile phone ≥5 hours per day. The hereditary factor we studied, the presence of spinal pain in participants' parents, was significantly associated with the presence of spinal pain in participants and the anatomic location of the pain. LBP and TP were more prevalent in participants with this hereditary factor present, whereas NP was more prevalent in participants without this specific hereditary factor.

According to a global study about occupational factors, published in 2005, 37% of LBP in the world is a result of the work environment as a risk factor, with a higher percentage in countries with lower health status [25]. NP was more common in participants with office (sedentary) jobs, and LBP in those with physical jobs [11]. In our study, there was no difference in the occurrences of NP, TP and LBP concerning the type of work, sedentary or physical, but employed persons and retired participants had a statistically significant risk of spinal pain compared to unemployed people. Considering the anatomical location of spinal pain, it is noticeable that employed persons had a higher prevalence of NP, compared to unemployed and retired people, while retired persons had a higher prevalence of LBP compared to employed and unemployed persons. In this study we did not study the type of occupation, so we could not stratify results based on different types of working environment.

Insufficient or excessive physical activity may be an important factor for functional spinal pain, especially in the growing period [26]. We found that all types of spinal pain together had a significant association with irregular physical activity but not with physical inactivity. Irregular physical activity was not a significant factor for NP, TP and LBP when this association was analyzed for these conditions individually.

This study had several limitations. We analyzed point prevalence and not lifetime prevalence, which is not necessarily comparable to other studies analyzing prevalence of spinal pain. The difficulty in comparing estimated prevalence due to research heterogeneity is a global problem because there is no uniform research methodology for such studies [11],[12]. We used online data collection as the simplest and most economically acceptable way to collect data. The limitation of this methodology may be reduced representativeness of the sample, as online data collection excludes access to individuals without internet access and potentially older individuals. However, to mitigate these issues as much as possible, we used a design weighted based on the number of inhabitants in the regions of BiH and their demographic characteristics (sex and age group). Moreover, a systematic review from 2016 reported similar representativeness of samples recruited on Facebook and known traditional methods [17].

Despite limitations, we believe that our study is an important contribution due to reported lack of data on the prevalence of spinal pain syndrome in our geographical region [14]. We were unable to find any spinal pain prevalence data in BiH, so this could be the first study on this topic in BiH and may serve as a comparison for future spinal pain prevalence studies that will be conducted in BiH. Moreover, this study can serve as a “wake-up call” and the beginning of changes in health policy and health approaches in our country, which need to be in line with scientific recommendations for painful conditions [27]–[30].

Future research in this area should aim to homogenize ways of determining the prevalence of spinal pain syndrome and associated risk factors, to enable comparison of data between different studies on pain prevalence. The problem of data heterogeneity in epidemiological research has been a problem for decades, which requires a global consensus about methodology recommendations, validated questionnaires and preferred statistical processing and interpretation of data.

## 5. Conclusions

To our knowledge, this is the first study of prevalence of spinal pain in Bosnia and Herzegovina. The prevalence of spinal pain in the surveyed adult BiH population in 2018 was very high. Demographic factors associated with spinal pain were sex and age 30 to 50 years. Behavioral factors associated with spinal pain include work status, irregular physical activity and length of watching TV per day. Some of the factors associated with spinal pain are modifiable, and therefore interventions for their elimination should be considered as a public health measure.
