# Supplementary material for: Prevalence of spinal pain in a population of Bosnia and Herzegovina
Source: AIMS Public Health. 2022 Dec 5;9(4):790–804. doi: 10.3934/publichealth.2022053 (PMC9807413; doi:10.3934/publichealth.2022053)
Supplement: Supplementary file 1 [file publichealth-09-04-053-s001.pdf]

Public Health

AIMS Public Health, 9(4): 790–804.
DOI: 10.3934/publichealth.2022053
Received: 20 June 2022
Revised: 11 November 2022
Accepted: 21 November 2022
Published: 05 December 2022

http://www.aimspress.com/journal/aimsph

Research article

Prevalence of spinal pain in a population of Bosnia and Herzegovina

Antonija Hrkać[1,*], Roberta Perković[1,2], Branko Krišto[2,3] and Livia Puljak[4]

[1] Faculty of Health Studies, University of Mostar, Mostar, Bosnia and Herzegovina
[2] Cantonal Hospital, Dr. Fra Mihovil Sučić, Livno, Bosnia and Herzegovina
[3] School of Medicine, University of Mostar, Mostar, Bosnia and Herzegovina
[4] Center for Evidence-Based Medicine and Health Care, Catholic University of Croatia, Zagreb, Croatia

* **Correspondence:** Email: antonija.hr@gmail.com; Tel: +38763408127; Fax: +38736337051.

Appendix 1. A more detailed description of the study methods

Inviting the participants

Potential participants received a personal message (cover letter) with a written invitation about the study and a link to the study. In both the cover letter and the introductory part of the questionnaire, participants were asked to forward the message and questionnaire to other individuals living in the BIH. We used “snowball sampling” by first contacting known contacts who were asked to forward a study web address with an available questionnaire to their contacts with different demographic characteristics in all regions of BIH. We asked contacts for information about the number of individuals that they have further contacted.

Pilot testing of the questionnaire

Pilot testing of the questionnaire for understanding, clarity, and relevance was conducted before the start of the survey (n = 50 participants with different sociodemographic characteristics). We revised the survey items based on the feedback from pilot testing. The survey took approximately 10 min to complete.

A detailed structure of the questionnaire

The full questionnaire used in this study is presented in the last section of this Appendix. The questionnaire consisted of 29 questions, open and closed-ended nominal, ordinal, and ratio/interval answers. The first 14 questions were about demographic characteristics (geographical region, age, sex, height, weight, education, work, smoking status (Yes/No), physical activity (Yes/No), intensity of physical activity (day/week), time spent (h/day) watching television and using computer and cell phone, history of spinal pain in parents) and 13 questions were about characteristics and consequences of spinal pain (presence, duration, back segment, type of pain, pain intensity, pain frequency, medical help, drug use, absence from work/school, frequency of absenteeism due to spinal pain). To avoid recall bias, we asked participants about their present pain and its duration. The point prevalence was measured. The presence of spinal pain was examined through the question "Do you have spinal pain". Participants who responded "yes" were considered to have spinal pain. Subjects who chose the upper back for localization of their pain were classified as neck pain; middle back as thoracic pain and lower back as LBP. The duration of spinal pain was classified as follows: the presence of pain symptoms for up to 6 weeks indicates acute pain, 6 to 12 weeks of sub-acute pain, and ≥12 weeks of chronic pain.

Pain intensity was measured via a numerical rating scale ranging from 0 to 10, with 0 indicating "no pain", and 10 indicating "worst pain". Pain intensity scores were converted into four categories: 0–1 no pain, 2–4 mild pain, 5–7 moderate pain, and 8–10 intensive pain. The question about "Diagnosed change" refers to identified changes in the spine, an intervertebral disc (IVD) hernia. The frequency of physical activity was studied with three answers that indicated specific frequency, and the fourth answer "I don't know, depends on the week" was considered irregular physical activity.

Data storage and handling

Only the principal investigator (AH) had access to the stored data; she monitored the data collection daily and re-contacted primary contacts from targeted regions with a request for the recruitment of further potential participants with the required demographic characteristics. Collected data were downloaded immediately after the study was completed, then removed and permanently deleted from the Internet.

Full version of the questionnaire used in the study

Spinal pain prevalence questionnaire

Dear Sir/Madam,

You are invited to participate in a study conducted to determine epidemiological data on the prevalence of spinal pain in the population of Bosnia and Herzegovina (BiH). Eligible study participants are inhabitants of BiH, of both sexes, aged 16 years and older. The questionnaire consists of 29 questions. The first 15 questions are related to risk factors that can lead to spinal pain. Other questions include information needed to determine the prevalence of the current spinal pain and the factors associated with it. The questionnaire is completely anonymous; you will not be asked to specify Your name date, place of birth, or place of residence (only canton). It takes on average 7 minutes to complete the questionnaire. Please read carefully and answer the questions honestly. Before completing the questionnaire, please read the Informed Consent outlining your rights.

Request: Please forward the accompanying message and the link on which the questionnaire can

be accessed to your contacts all over BiH. Thank you!

Informed Consent: Participation in the study is voluntary and completely anonymous. Participation as well as refusal to participate does not and will have no consequences on you and your health. The collected personal data will remain known only to the researchers; they will serve to determine the epidemiological data in this study and will not be used for other purposes. Filling in and submitting the questionnaire below will be considered as your acceptance of participating in the study. Thank you in advance!

If you have read your rights related to participation in this study and if you agree with the above and would like to participate in this research, please confirm this by entering the study questions.

1. Your Canton in Bosnia and Herzegovina
o Herzegovina-Neretva Canton
o West Herzegovina Canton
o Canton of Livno
o Middle Bosnian Canton
o Sarajevo Canton
o Canton of Tuzla
o Una-Sana Canton
o Zenica Doboj Canton
o Bosnian Podrinje Canton
o Republic of Serbian
o Brcko District

2. Your age is… (Enter years) ______________________

3. Sex
o Men
o Women

4. Your body weight (in kg) ___________________

5. Your body height (in cm) ___________________

6. Your educational status (Select one answer)
o Student in High school/Student on College
o Elementary school
o High school
o College

7. Your work status (Select one answer)
o Student in high school/Student on college Employed
o Unemployed
o Retired

8. Type of work You are performing
o Physical work
o Sedentary (office) work
9. Do You smoke?
o Yes
o No

10. Are You physically active?
o Yes
o No

11. The frequency of Your physical activity per week is (Select one answer)
o 1 to 2 days a week
o 3 to 4 days a week
o 5 to 6 days a week
o I don't know. it depends on the week

12. How much time do You spend in one day watching TV?
o 1 to 2 hours a day
o 2 to 3 hours a day
o 4 to 5 hours a day
o More than 5 hours a day

13. How much time do You spend on your computer / Mobile phone a day?
o 1 to 2 hours a day
o 3 to 4 hours a day
o 5 to 6 hours a day
o 7 hours and more a day

14. Do Your parents had (or have) spinal pain?
o Yes
o No

15. Do You have spinal pain?
o Yes
o No

16. How long do You have spinal pain?
o Up to 6 weeks
o From 6 to 12 weeks
o 12 weeks and over
o I have no spinal pain

17. In which part of Your back do you feel pain?

- o Upper Back (Neck, Head)
- o Middle back
- o Lower back

18. Please choose a claim describing Your spinal pain
o My pain remains in the back (neck, scapula, middle and lower back).
o My pain spreads to the head, neck, arms and legs.

19. Do you have any diagnosed changes to Your spine?
o Yes
o No

20. My spinal pain is present (Choose how often)
o Daily
o Once a week
o Once a month
o After heavy physical activity

21. On a scale of 0 to 10 where 0 indicates no pain and 10 the worst possible pain. which grade would best describe Your pain? (Enter number) ________________
22. Are you taking any medications (analgesics) against spinal pain?
o Yes
o No

23. How often do you take your spinal pain medication?
o Daily
o Once a week
o Once a month
o Very rare
o I don't take medication

24. Have you sought medical attention for your spinal pain?
o Yes
o No

25. Where? (Refers to seeking medical help)
o General practitioner / family medicine
o Neurologist / Neurosurgeons
o Physical therapist
o Some others

26. Have you been absent from work or school / college because of your spinal pain?
o Yes
o No

27. How many days? (Refers to absences from work and / or from school. college)
- o 1 to 2 days
- o 3 to 4 days
- o 5 to 7 days
- o More than a week
- o I was not missing

28. Are you familiar with the proper postures and body movements that aim to protect our spine?
- o Yes
- o No

29. If so. do you apply the same in your daily life activities?
- o Yes
- o No
- o I don't know

Please click on SUBMIT to send your anonymous responses.

Once again. Thanks for Your participation!

AIMS Press

© 2022 the Author(s), licensee AIMS Press. This is an open access article distributed under the terms of the Creative Commons Attribution License (http://creativecommons.org/licenses/by/4.0).
